# Bioinspired Conductive Enhanced Polyurethane Ionic Skin as Reliable Multifunctional Sensors

**DOI:** 10.1002/advs.202300857

**Published:** 2023-04-24

**Authors:** Bicheng Zhao, Jiaqi Yan, Fen Long, Wu Qiu, Guoqing Meng, Zhicheng Zeng, Hui Huang, Han Wang, Naibo Lin, Xiang‐Yang Liu

**Affiliations:** ^1^ Research Institution for Biomimetics and Soft Matter The Higher Educational Key Laboratory for Biomedical Engineering of Fujian Province Research Center of Biomedical Engineering of Xiamen Department of Biomaterials College of Materials The State Key Laboratory of Marine Environmental Science (MEL) College of Ocean and Earth Sciences Shenzhen Research Institute of Xiamen University Xiamen University 422 Siming Nan Road Xiamen 361005 People's Republic of China; ^2^ Printed Intelligent Device Group Singapore Institute of Manufacturing Technology (SIMTech) Agency for Science Technology and Research (A*STAR) Singapore 636732 Republic of Singapore; ^3^ Selangor Sepang A1‐476 Xiamen University Malaysia Jalan Sunsuria 43900 Federation of Malaysia

**Keywords:** bioinspired, ionic conductivity, ionogels, multifunction sensor

## Abstract

Ionogels prepared from ionic liquid (IL) have the characteristics of nonevaporation and stable performance relative to traditional hydrogels. However, the conductivities of commonly used ionogels are at very low relative to traditional hydrogels because the large sizes of the cation and anion in an IL impedes ion migration in polymer networks. In this study, ultradurable ionogels with suitable mechanical properties and high conductivities are prepared by impregnating IL into a safe, environmentally friendly water‐based polyurethane (WPU) network by mimicking the ion transport channels in the phospholipid bilayer of the cell membrane. The increase in electrical conductivity is attributed to the introduction of carboxylic acid in the hard segment of WPU; this phenomenon regularly arranges hard segment structural domains by hydrogen bonding, forming ionic conduction channels. The conductivities of their ionogels are >28–39 mS cm^−1^. These ionogels have adjustable mechanical properties that make the Young's modulus value (0.1–0.6 MPa) similar to that of natural skin. The strain sensor has an ultrahigh sensitivity that ranges from 0.99 to 1.35, with a wide sensing range of 0.1%–200%. The findings are promising for various ionotronics requiring environmental stability and high conductivity characteristics.

## Graphical abstract

Polyurethane‐based ionogels are synthesized for multifunctional human signal sensing, inspired by cell membrane ion channels. Ionogels that efficiently accommodate ionic liquids have ideal mechanical properties to fit tissue while retaining their high conductivities of 39 mS cm^−1^. The strain and pressure sensors fabricated with the ionogels have ultrahigh sensitivity and stability characteristics. Moreover, ionogels obtain electrophysiological signals when used as conductive gels.

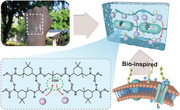

## 1 Introduction

In the last decade, the increasing interest in human–computer interactions and health monitoring has accelerated the development of flexible electronics.^[^ ^1^, ^2^, ^3^ ^]^ In the early days of this field, metal, and graphite conductive electrodes were used to prepare stretchable flexible electronic materials with flexible polymer elastomers, ensuring relatively high conductivity; however, their flexibility, sensing sensitivity, and range are relatively low, which is not suitable for monitoring human motion behaviors over a wide range.^[^ ^4^, ^5^, ^6^, ^7^ ^]^ Afterward, hydrogels became a new research topic for scientists as a new generation of flexible conductive materials.^[^ ^8^, ^9^, ^10^, ^11^, ^12^ ^]^ This kind of material is soft and has a wide detection range. For organisms, mechanoreceptors release transmembrane Donnan potential through mechanical gating; ionogels change their physical properties through pressure drive. Hydrogels take ions as charge carriers; this phenomenon is similar to human nerve signal conduction.^[^ ^13^, ^14^ ^]^ However, the solvent of hydrogels is easy to volatilize, causing unstable shortcomings, such as electrical signal baseline that drifts over time.^[^ ^15^ ^]^

Recently, there have been gels (or ionogels) made with ionic liquids (IL) in polymer networks without aqueous solvents.^[^ ^16^, ^17^, ^18^, ^19^ ^]^ An IL is a salt completely composed of cations and anions in liquid form at or near room temperature. IL substituents have high steric hindrance, preventing ions from stacking regularly. Relative to hydrogels, ionogels have nonvolatile properties and chemical and heat stability properties.^[^ ^6^, ^11^, ^12^, ^13^, ^14^ ^]^ Ionogels have low conductivities because various charged carriers have large volumes and are easily hindered by the polymer matrix.^[^ ^20^ ^]^ A large amount of evidence has shown that in typical polymer ionic conductor systems, the dominant ion motion occurs in the amorphous region through local sectional motion.^[^ ^21^, ^22^ ^]^ Carrier mobility is strongly linked to the glass transition of the polymer in the amorphous state. A low glass transition temperature (*T_g_*) usually results in conductive enhancement.^[^ ^22^ ^]^ The polymer must have an amorphous structure at room temperature (or working temperature), or it must operate around its crystalline melting point to achieve a liquid‐like microscopic environment.

However, it is difficult to use a single polymer with a low *T_g_* as a substrate for ionogels. These low *T_g_* polymers make the whole system appear as a viscous liquid when they swell at room temperature; the ionogels in this state do not maintain their shape or have stretchability. To date, physical blending, cross‐linking, or copolymerization are used to prepare the polymer matrices of ionogels to improve mechanical properties and conductivities.^[^ ^23^, ^24^ ^]^ Polyurethane is composed of a soft and hard segment. The soft segment refers to the polyol part in which the *T_g_* is lower than room temperature, giving the polyurethane better flexibility. The hard segment formed by the reaction of isocyanate and small molecule chain extender has high rigidity, strong polarity and high *T_g_*, endowing the polyurethane with certain strength and hardness characteristics.^[^ ^25^, ^26^, ^27^ ^]^ Due to the thermodynamic incompatibility between soft and hard segments, polyurethane materials tend to aggregate and form independent microregions; this phenomenon presents a unique microphase separation structure. In this special structure, the hard segment region acts as a physical cross‐linking point to connect the polyurethane chain segments together. According to dissipation‐induced toughening theory,^[^ ^17^, ^28^, ^29^, ^30^, ^31^, ^32^ ^]^ the hard segment of polyurethane connecting the polyurethane polymer chains dissipates energy during deformation and promotes toughness. However, due to the high *T_g_* and ion incompatibility, the hard segment structural domain of polyurethane hinders ion migration and reduces the conductivity.^[^ ^33^ ^]^ According to the theory proposed previously, the conductivity and mechanical strength of the gel are in conflict with each other. this conflict appears even more pronounced for IL with large steric hindrance. A type of mechanically robust ionogels have reinforced conductivity by introducing lithium ions that can migrate in the hard segment structural domain.^[^ ^18^ ^]^ In this article, by introducing ion exchange sites into the forbidden zone of ion movement (the hard domain of polyurethane), the conductivity of the gel significantly improves, and solid formation of the gel is ensured.

**Figure 1:**
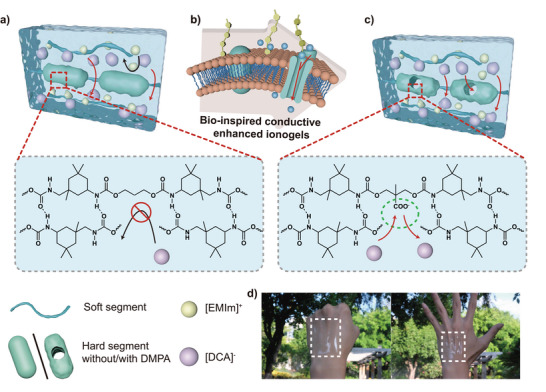
Schematic illustration of the principle of conductive enhanced ion gels inspired by cell membrane ion channels. a) Hard domain of WPU without dihydroxy‐methylpropionic acid (DMPA) doping severely affects ion migration in the gel. b) We were inspired by the fact that ion channels in the cell membrane allow for ion migration. c) A carboxyl group was introduced into the hard domain of WPU to provide an ion migration site and effectively enhance ion migration in the gel. d) Ionogel physical picture (ionogels are attached to the back of the hand, and they stay attached with skin movement).

In the principle of the ion pathway on biological cell membranes, a pure phospholipid bilayer does not allow ions to pass through. Most of the amino acid side groups of sodium ion channel proteins contain carboxyl groups. These groups negatively charge the surfaces of their channels; thus, sodium ions are repeatedly combined and released, and sodium ions enter cells under an ion concentration gradient (**Figure** **1**b). Inspired by ion channel proteins, we introduce carboxyl groups into the hard segment domain of polyurethane to promote conductivity; the artificial ion exchange sites of carboxyl groups endow the smooth passage of ions through the hard segment of polyurethane under an electric field (Figure 1a,c). The prepared polyurethane ionogels have high ionic conductivities of 37 mS cm^−1^; the tensile stress is 0.2 MPa and the strain is 200%, generating fast and repeatable sensing signals over a wide strain range. The high ionic conductivity opens more potential applications for ionogels in the future.

## 2 Results and Discussion

In this work, transparent ionogels with high ionic conductivities are achieved by uniformly distributing an IL of 1‐ethyl‐3‐methyl‐imidazold‐dicyandiamide ([EMIm][DCA]) in a polymerization network formed by green and nontoxic waterborne polyurethane (WPU). The hydrogen bond formed between WPU are adjusted by changing the content of [EMIm][DCA] to obtain ionogels with modulus values similar to those of human skin. WPU is synthesized in three steps (Scheme S1, Supporting Information). First, a linear WPU prepolymer is synthesized by a condensation reaction of polytetrahydrofuran (PTMG) diol, polydimethylsiloxane (PDMS) diol, isoflurone diisocyanate and dihydroxy‐methylpropionic acid (DMPA). Second, 1,4‐butandiol (BDO) as a chain extender and 2‐hydroxy‐ethyl acrylate (HEMA) as an end‐capping reagent are added to the WPU prepolymer to form a WPU polymer with a double‐bonded end. Finally, the polymer with a double bond closure is subjected to radical polymerization to form a WPU solution (30 wt.%) with a cross‐linked structure. A WPU network consists of PTMG and PDMS soft and hard segments, including diisocyanate and a chain extender. The structure of the WPU is verified by Fourier transform infrared spectroscopy (Figure S2, Supporting Information). [EMIm][DCA] has high electrochemical stability and is intersoluble with water in various proportions. The aqueous solution of WPU is mixed with the IL [EMIm][DCA] solution (30 wt.%) and cast on a polytetrafluoroethylene (PTFE) substrate; this process is followed by solvent evaporation at 25 °C and a humidity of 60% (Scheme S2, Supporting Information). As the solvent evaporates, the nonvolatile IL and WPU gradually and slowly form ionogels, named WPU/IL*_x_* (*x* is the mass ratio of IL:WPU).

**Figure 2:**
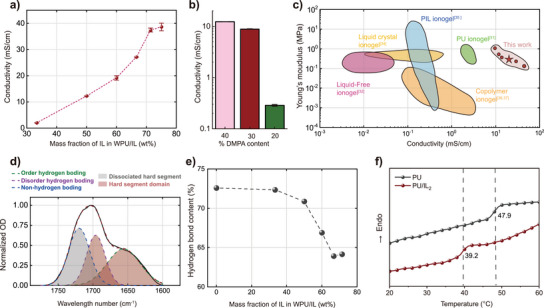
a) Conductivities of WPU/IL*_x_* with various IL contents (x represents the mass ratio of PU and IL, *x* = 0.5 to 3.0). b) Influences of different DMPA contents on electrical conductivities. c) Comparison of conductivity and modulus values with other ionogels. d) Peak deconvolution of WPU/IL_2.0_. e) Hydrogen bond contents in the WPU/IL*_x_* values of samples. f) DSC curves of WPU and WPU/IL_2.0_ ionogels.

Figures 1d and S1a, Supporting Information, show photographs of WPU/IL_2.0_ ionogels. To systematically discuss the correlations between the structures and properties of the WPU/IL*_x_* ionogels, we characterized the chemical structures of the WPU, IL, and ionogels by Fourier transform infrared (FTIR) spectroscopy (Figure S3, Supporting Information). The absorption peak at 1026 cm^−1^ corresponds to the vibration absorption of Si—O–Si, the absorption peak at 1700 cm^−1^ represents the vibration absorption of C≐O, and the peak at 2126 cm^−1^ corresponds to the anion symmetric stretching vibration of [DCA]^−^. These peaks appear in the infrared absorption spectra of WPU/IL_2.0_ simultaneously, indicating that WPU and IL successfully mix. To explore the strong interactions of hydrogen bonding between WPU and IL, carbonyl (CO) vibration absorptions of FTIR between 1580–1775 cm^−1^ on the WPU molecules are conducted for peak splitting (**Figure** **2**d,e, and Figure S3, Supporting Information).^[^ ^25^ ^]^ Through deconvolution peak splitting, we obtain a C≐O ordered hydrogen bond vibration absorption peak at 1600 cm^−1^, a C≐O disordered hydrogen bond vibration absorption peak at 1700 cm^−1^ and a C≐O nonhydrogen bond vibration absorption peak at 1722 cm^−1^. The area of each absorption peak corresponds to the proportion of different vibration absorption modes. With increasing IL content, the ratio of the vibration absorption peak of the C≐O nonhydrogen bond gradually increases, potentially reducing the hard segment domain content.^[^ ^24^ ^]^

The ionogel conductivities with different IL contents are measured (Figure 2a). With increasing IL content, the conductivities of the ionogels first increase from 2 to 39 mS cm^−1^. To investigate whether the introduced carboxyl groups in the hard segment domain of WPU have the same effects as ion channels in the cell membrane. By reducing the DMPA contents to different values, a series of WPUs are synthesized (Figure 2b), and the conductivities gradually increase with increasing DMPA hard segments. This result suggests that IL ions pass through the incompatible hard segment domain to improve the ionic conductivity. The conductivity of the enhanced ionogel is much higher than that of all of the ionic gel diagrams that have been previously reported (Figure 2c). We have tested the alternating current (AC) impedance of the ionogels (Figure S12, Supporting Information), and its Nyquist plot conforms to the electrochemical behaviors of traditional gels. The semicircular arc in the high‐frequency region is small, indicating that the impedances between the ionogels and the contact faces are low.

To explore the ionic interactions between WPU and IL, the mixed emulsions of WPU and IL are measured by a dynamic light scattering (DLS) particle size analyzer (Figure S4, Supporting Information). With increasing IL content, the particle size of the WPU colloid gradually increases, and the state of the WPU colloid changes from stable to unstable. In DLOV theory, the interactions between two latex particles are considered the sum of the van der Waals attraction and electrostatic repulsion characteristics. Ion binding between the IL liquid and carboxyl group on WPU reduces the electrostatic repulsion of WPU and the net forces between latex particles.

X‐ray diffraction (XRD) data of WPU/IL_2.0_ and WPU only show broad peaks at 20°; these findings are consistent with the XRD data of amorphous polymers, indicating that WPU/IL_2.0_ ionogels are amorphous and IL is uniformly dispersed in WPU without crystallization (Figure S8, Supporting Information).^[^ ^34^ ^]^ Relative to the XRD pattern of pure WPU, the peak of WPU/IL_2.0_ is wider, indicating that the content of the hard segment domain is lower. Due to the amorphous structure, WPU/IL_2.0_ ionogels are optically transparent (Figure S1a, Supporting Information). By measuring the optical losses of WPU/IL*_x_* ionogels in different proportions (Figure S9, Supporting Information), the gel transparency increases with increasing IL, which is evidence that IL opens the hydrogen bond structure of the WPU hard segment.

The *T_g_* values of WPU and WPU/IL_2.0_ ionogels are measured by differential scanning calorimetry (DSC) to further study the interaction between WPU and IL. Both WPU and WPU/IL_2.0_ have *T_g_* values of 20—60 °C; this phenomenon is attributed to the hard segment of WPU (Figure 2f). When the concentration of IL increases, *T_g_* shifts to a low value, indicating that the presence of IL impedes the formation of hydrogen bonds between WPU hard segments and regularly stacks the hard segments.^[^ ^31^ ^]^ The low shift in the *T_g_* of the WPU hard segment is consistent with the results obtained by peak splitting in the FTIR data.

To characterize the temperature stability and nonvolatilization characteristics of ionogels, the influences of temperature on the conductivities of ionogels are investigated using an oven and freezer layer (Figure S5, Supporting Information). The resistance value of WPU/IL_2.0_ is almost stable at ≈22 kΩ from −25 °C to 80 °C; however, the resistance value increases to 34 kΩ near −20 °C because IL is stable over a wide temperature range. Nevertheless, the melting points of [EMIm][DCA] at −5 °C and −20 °C limit the migration of [EMIm][DCA] inside the ionogel. The influence of humidity on ionogels is also explored, we find that humidity affects the stability of the resistance of ionogels (Figure S6, Supporting Information). With the increase of humidity, the resistance value of the ionogels gradually decreases, which means that its conductivity is gradually increasing. To investigate the stability levels of ionogels in air, we compare the ionogels with some of the common conductive hydrogels by weightlessness tests (Figure S7, Supporting Information). After 10 days of exposure to an external atmosphere environment (25 °C, 65 RH%), WPU/IL_2.0_ does not absorb volatile or water components; polypyrrole (PPy), silk fibroin, and polyacrylonitrile (PAN) hydrogels lose all their moisture and conductive functions after one day.

**Figure 3:**
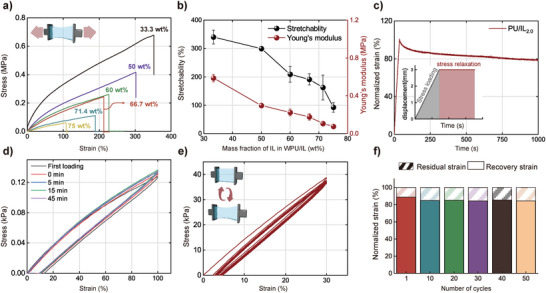
a) Stress–strain curves. b) Young's modulus and stretchability of WPU/IL*_x_*. c) Mechanical relaxation profile of WPU/IL_2.0_ after 100% strain; the inset is the tensile displacement‒time curve during stress loading and relaxation. d) Stress recovery curve of WPU/IL_2.0_. e) Typical tensile stress–strain curves of WPU/IL_2.0_. f) Residual strain and recovery ratio plotted versus the loading/unloading cycles.

The mechanical properties of WPU/IL*_x_* are measured, and the tensile fracture toughness strength and Young's modulus values of the sample are extracted from the stress‒strain curves (**Figure** **3**a, Figure S10, Supporting Information). With increasing IL concentration, the elongation at break and Young's modulus decrease gradually (Figure 3b). This phenomenon occurs because the IL reduces the content of the WPU hard segment domain. The WPU/IL_2.0_ ionogels have Young's modulus values between 0.075 and 0.586 MPa; this range is similar to that of human skin. Additionally, the ionogels have elongation at break values of 200%. Mechanical relaxation experiments (Figure 3c), stress recovery curves (Figure 3d), and stress cycle curves at 30% strain (Figure 3e, f) are performed on WPU/IL_2.0_ under 100% strain to simulate various states of the ionogel working on the human surface. From Figure 3c, the ionogel retains 80% of its stress over a long period (1000 s) of tension; this phenomenon indicates that the ionogel maintains its shape for a long time without relaxation when deformed by human movement.

The stress recovery curve is completely restored to the original curve after a relatively short period after a large deformation of 100% (Figure 3d). WPU/IL_2.0_ ionogels’ cyclic tensile tests were carried out at high strain (100%) in 50‐cycle curve (Figure S11, Supporting Information), it's ≈50% strain lag after 50th cycle, and the ionogels can restore to its original state by taking a short break. And the loading and unloading curves of WPU/IL_2.0_ under low strain (30%) overlap and show a negligible lag (Figure 3e). The excellent mechanical reversibility of WPU/IL_2.0_ is quantitatively represented by the low residual strain and high recovery rate from the curves of Figure 3e; a low residual strain level (15%) corresponding to a recovery rate of 75% is observed in 50 consecutive cycles (Figure 3f). Rapid deformation and recovery at low strain (30%) are ideal and important properties for sensory and drive applications, such as artificial muscle skin‐like sensors and tissue–electronic interfaces.

**Figure 4:**
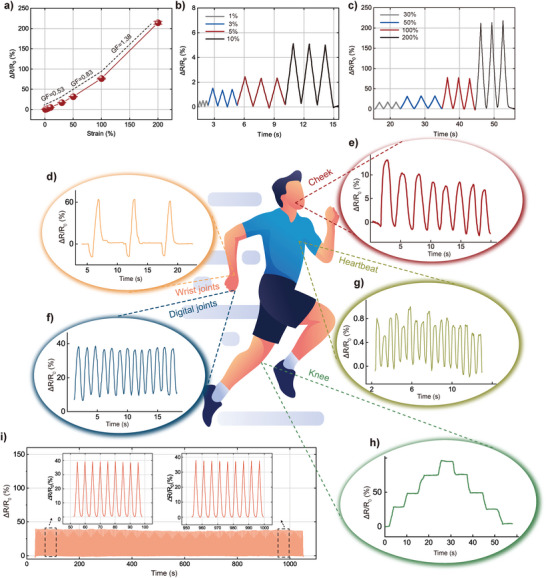
Relative resistance changes of WPU/IL_2.0_ I‐skins (strain sensor) as a function of a) strain, b) under small strains (3, 5, and 10%), and c) under large strains (30, 50, and 100%). d) I‐skin fixed on a wrist that rotates back and forth. e) Facial movement during breathing. f) Repeated bending/unbending movements of a finger. g) Heartbeats. h) I‐skin fixed on a leg joint bent at different angles. i) Cyclic stability tests of I‐skin under 30% strain for 200 cycles.

Recently, soft and flexible skin‐like electronic devices have received increasing attention as a novel platform for integration with soft tissues for human–computer interactions, health monitoring, and medical treatment.^[^ ^36^, ^37^ ^]^ Ionic skin (I‐skin) is an ionotronic device in which ions replace electrons as the carrier of electrical signal transmission to simulate the sensing function of natural skin. From the mechanical properties of WPU/IL_x_, the modulus of WPU/IL_2.0_ is similar to that of the human epidermis, and the strain elongation rate of 200% is sufficient for monitoring all parts of the human body,^[^ ^1^ ^]^ while its conductivity is also at a high level. **Figure** **4**a shows the relative change in the resistance (∆*R*/*R*_0_) values of WPU/IL_2.0_ ionogels as an I‐skin function of strain. The gauge factor (GF) values of strain sensors are the slopes of the strain sensors (∆*R*/*R*_0_) relative to the strain curve; this phenomenon is an important parameter for measuring the sensitivity of a strain sensor.

The detection range of our ionogels is from 1% to 200%, and the detection range monitors all parts of human motion. In the small strain range (1% to 30%), the GF of the ionogel is 0.53. GF is 0.83 in the medium strain range of 30% to 100%. In the large strain range (100% to 200%), the GF of the ionogel is 1.38. Sensitivity is the most important sensor indicator. The sensitivity of the simplest piezoresistive sensor is described by Equation (1),

(1) $$\frac{dR}{R}=\left(1+2\mu \right)\;\varepsilon -\frac{d\sigma }{\sigma }$$

where *R* is the resistance, *σ* is the ionic conductivity, *µ* is Poisson's ratio, and *ε* is the strain. From the equation, the sensitivity is related to the Poisson's ratio and the ionic conductivity of the material. We calculated the conductivities of the ionogels under dynamic conditions (Figure S13, Supporting Information) and Poisson's ratios (Table S1, Supporting Information). The µ of WPU/IL_2.0_ ionogels equals 0.97. During stretching, the conductivities of the ionogels gradually increase. Ionogels may generate orientation during stretching to form high‐speed channels for ion transfer.^[^ ^34^ ^]^ Therefore, the GFs of the ionogels are affected by both the deformation and conductivity.

From the equation, we can find that the resistivity change rate (*dR*/*R*) is negatively correlated with the conductivity change rate (*dσ*), and the conductivity of WPU/IL_2.0_ increases with the strain increase (Figure S13, Supporting Information), which will reduce the GF of the sensor. To obtain higher GF, the effect of the conductivity change rate can be reduced by increasing the initial conductivity (*σ*). WPU/IL_0.5–2.0_ ionogels were fabricated as strain sensors for the measurement of GF (Figure S14, Supporting Information). It can be found that their GF decreases continuously with the decrease of conductivity, while the GF of WPU/IL_0.5_ ionogels shows a negative value, which indicates that the change rate of conductivity is much higher than that of strain. This shows that increasing the conductivity of the polyurethane ionogel helps to increase its GF. In addition, the ionogels have a low detection limit (0.68%), even when monitoring the human heartbeat (Figure 4g). This phenomenon suggests that WPU/IL_2.0_ ionogels have the potential to be used as I‐skin. The WPU/IL_2.0_ ionogels produce a stable ∆*R*/*R*_0_ signal during cyclic stretching as the mechanical strain gradually increases from 1% to 200%, indicating their high monitoring sensitivity and reliability (Figure 4b,c). Figure 4i shows the ∆*R*/*R*_0_ signal of the I‐skin at 30% strain over 200 uninterrupted extension–release cycles. During expansion, the intensity of the ∆*R*/*R*_0_ signal is 38%, and the reduction is negligible after 200 extension–release cycles. This finding indicates that WPU/IL_2.0_ ionogels have ultradurable and sensitive sensing performance levels as I‐skin. Next, WPU/IL_2.0_ ionogels are used as wearable ionotronic devices to monitor various human movements. Figure 4d shows the signal of the wrist flip motion. Figure 4e shows the agitation of the cheeks during breathing. Figure 4f shows the change in the *R*/*R*_0_ signal while gradually bending a straight finger. Figure 4h shows the changes in the *R*/*R*_0_ signal when the knee is bent. The *R*/*R*_0_ signal increases with increasing knee bending angle. When the knee bending angle is constant, the *R*/*R*_0_ signal remains unchanged. Due to the good resilience characteristics of the ionogels, the *R*/*R*_0_ signal returns to its original value when the knee straightens again. As shown in Figure 4h, I‐Skin monitors multiple cycles of wrist back‐and‐forth motion in real time; there is no lag in *R*/*R*_0_ changes whether the ionogels are stretched or compressed. In addition, I‐Skin monitors very small human activities, such as heartbeats. When the I‐skin is attached to the left chest with commercial tape, the I‐skin generates a repeatable and reversible *R*/*R*_0_ signal (Figure 4g).

**Figure 5:**
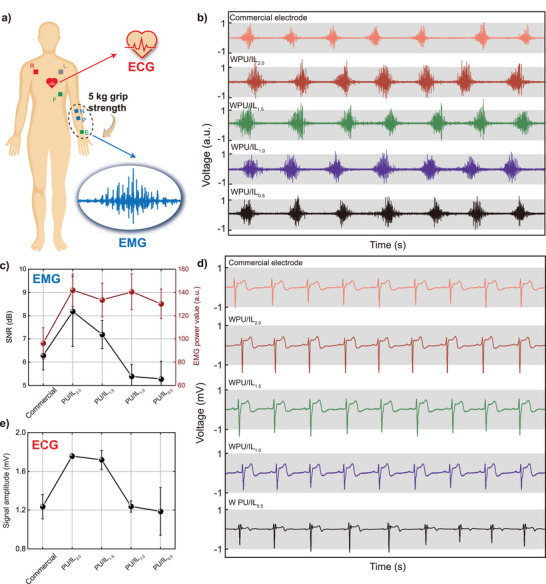
WPU/IL*_x_* ionogels are used for EMG and ECG monitoring. a) EMG and ECG signal acquisition diagram. b) EMG and d) ECG acquisition contrast diagram of the WPU/IL*_x_* ionogels and commercial electrode. c) SNR and power values of commercial and WPU/IL*_x_* ionogels electrodes in EMG. e) Signal amplitude of commercial and WPU/IL*_x_* ionogels electrodes in ECG.

In addition, the WPU/IL_2.0_ ionogels were used as conductive gels to obtain electrophysiological signals. **Figure** **5**a shows the applications of the WPU/IL_2.0_ ionogels in electromyography (EMG) and electrocardiograph (ECG). EMG and ECG signals were collected by BiosiganlsPULX Researcher, and the volunteer is fully voluntary and aware of any risks involved. Low‐modulus and high‐conductivity electrodes fit well to the skin surface and conduct electrophysiological signals, these characteristics are key to achieving a high signal‐to‐noise ratio (SNR) in electrophysiological measurements. To compare commercial and WPU/IL*_x_* ionogel electrodes, we collect the EMG signals of the forearm at 5 kg grip strength (Figure 5b). Through calculation, ionogels electrode has higher power value. The ionogel electrode of WPU/IL_2_ also has the highest SNR which is 8.2.

With the decrease of electrical conductivity, the SNR gradually decreases to 5.3. The SNR of a commercial electrode is 6.3. Commercial and WPU/IL*_x_* ionogel electrodes were used to collect the ECG signals (Figure 5d). As the IL content decreases, the collected ECG signals become more ambiguous. The difference between the highest value and the lowest value of the ECG signal is taken as the signal amplitude (Figure 5e). WPU/IL_2.0_ electrode can collect the strongest ECG signal. This is due to the high conductivity of WPU/IL_2.0_ ionogels.

**Figure 6:**
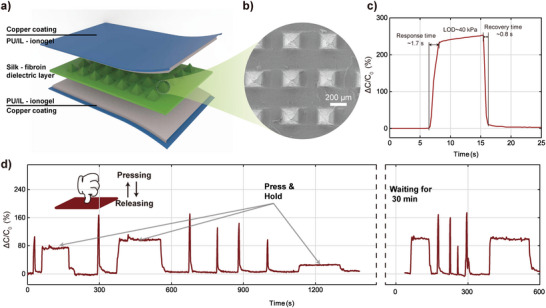
a) Model diagram of a capacitive pressure sensor. b) Micrograph of the silk fibroin‐based dielectric layer. c) Sensor signal when a 200 g weight (40 kPa) is pressed. d) Sensor signal diagram of frequent pressing by hand.

To show the multifunctional characteristics of our ionogels, a capacitive pressure sensor is prepared, and the principle diagram describes the design principle of the piezoelectric capacitive pressure sensor. The sensor features a sandwich structure of WPU/IL_2.0_ ionogels, silk fibroin microneedle, and ionogels.^[^ ^38^ ^]^ The thickness of the whole sensor is ≈2 mm (**Figure** **6**a). The optical microscopic image of the dielectric layer shows that the pyramidal array microstructures on the surface of the silk fibroin film are 600 µm high, 200 µm wide, and 500 µm apart (Figure 6b). When pressure is applied, the two gel electrodes move close to each other to change the capacitance value. When the capacitive pressure sensor is pressed with a 200 g weight (≈40 kPa), the response time is 1.7 s and the recovery time is 0.8 s. This result shows that the capacitive sensor has a fast response time and responds to pressure in a timely and sensitive manner (Figure 6c). This capacitive sensor is applied to monitor a range of intermittent stimuli; this sensor has a quick deformation response, a quick recovery after force release, a short response time, and a short recovery time (Figure 6d).

## 3 Conclusion

In summary, we have designed and efficiently prepared conductive ionogels. We introduce carboxyl groups into the hard segment domain of WPU to reduce the obstruction of the WPU hard segment domain on ionic migration by mimicking the principle of the cell membrane ion channel, increasing the ion conductivity (28–39 mS cm^−1^). Moreover, we find that the increase in IL content reduces the contents of hydrogen bonds and hard segment domains in WPU. As a result, for WPU ionogels, the ion conductivities increase and the moduli decrease (0.6–0.1 MPa), exhibiting mechanical properties similar to those of human skin. Using IL as solvents enable ionogels to remain stable over a long period relative to conventional hydrogels. The ionogels have an ultrahigh sensitivity ranging from 0.99 to 1.35, with a wide sensing range from 0.1 to 200%. In addition, strain testing of the ionogels shows that the ionogels have almost no hysteresis and creep, allowing the accurate monitoring of various human movements. Moreover, there is no solvent volatilization after long‐term exposure to air, and it works for a long period. Due to the ultrahigh conductivities of ionogels, their potential applications as conductive layers and flexible electrodes for capacitive sensors is demonstrated. This work provides new insights and practical methods for the molecular design and fabrication of ionogel polymer matrices, with broad application prospects in wearable electronics, biomedicine, and artificial intelligence.

## Conflict of Interest

The authors declare no conflict of interest.

## Supporting information

Supporting Information

## Data Availability

Research data are not shared.
