# Supplementary material for: Bioinspired Conductive Enhanced Polyurethane Ionic Skin as Reliable Multifunctional Sensors
Source: Adv Sci (Weinh). 2023 Apr 24;10(19):2300857. doi: 10.1002/advs.202300857 (PMC10323669; doi:10.1002/advs.202300857)
Supplement: Supplementary file 1 — Supporting Information [file ADVS-10-2300857-s001.pdf]

ADVANCED SCIENCE
Open Access

Supporting Information

for *Adv. Sci.*, DOI 10.1002/advs.202300857

Bioinspired Conductive Enhanced Polyurethane Ionic Skin as Reliable Multifunctional Sensors

Bicheng Zhao, Jiaqi Yan, Fen Long, Wu Qiu, Guoqing Meng, Zhicheng Zeng, Hui Huang, Han Wang, Naibo Lin and Xiang-Yang Liu**

Supporting Information

Bioinspired conductive enhanced polyurethane ionic skin as reliable multifunctional sensors

Bicheng Zhao, Jiaqi Yan, Fen Long, Wu Qiu, Guoqing Meng, Zhicheng Zeng, Hui Huang, Han Wang, Naibo Lin,* Xiang-Yang Liu*

B. C. Zhao, J. Q. Yan, F. Long, G. Q. Meng, Z. C. Zeng, Prof. N. B. Lin, Prof. X. Y. Liu

Research Institution for Biomimetics and Soft Matter, The Higher Educational Key Laboratory for Biomedical Engineering of Fujian Province, Research Center of Biomedical Engineering of Xiamen, Department of Biomaterials, College of Materials, The State Key Laboratory of Marine Environmental Science (MEL), College of Ocean and Earth Sciences, Shenzhen Research Institute of Xiamen University, Xiamen University, 422 Siming Nan Road, Xiamen 361005, People's Republic of China
Prof. H. Huang
Printed Intelligent Device Group, Singapore Institute of Manufacturing Technology (SIMTech), Agency for Science, Technology and Research (A*STAR), Singapore, 636732, Republic of Singapore
Prof. H. Wang
Selangor, Sepang A1-476, Xiamen University Malaysia, Jalan Sunsuria, 43900, Federation of Malaysia
*corresponding author
E-mail: linnaibo@xmu.edu.cn; liuxy@xmu.edu.cn

Experimental Section

Materials:

Polytetramethylene ether glycol (PTMG, M_w ~2000), isophorone diisocyanate (IPDI, 99%), N, N-dimethylformamide (DMF, AR), dibutyltin dilaurate (DBTDL, AR) and dimethylolpropionic acid (DMPA, AR) were purchased from Macklin. 2-hydroxyethyl methacrylate (HEMA, AR) was purchased from Aladdin. 1,4-Butanediol (1,4-BDO, AR) and triethylamine (TEA, AR) were purchased from Xilong Scientific. Polydimethylsiloxane (PDMS) diol (M_w ~10000) was purchased from Dow Corning. Ammonium persulfate (APS,

AR) was obtained from Sinopharm. 1-Ethyl-3-methyl imidazo ([EMIM][DCA]) was obtained from Dibo. PTMG and DMPA were dehydrated and dried in a vacuum drying oven at 120 °C for 4 h and then placed in a rapid glass dryer for later use. Molecular sieves (4 Å) were dried in a vacuum drying oven at 150 °C for 4 h. IPDI, 1,4-BDO, TEA, DBTDL and PDMS diol were dried by the 4 Å molecular sieves.

Preparation of waterborne polyurethane (WPU):

Dried IPDI (8.89 g, 0.04 mol), PTMG (20 g, 0.01 mol), and PDMS diol (0.89 g, 0.089 mmol) were added into a four-neck flask, and the mixture was stirred at 80 °C under a N_2 atmosphere. Then, DBTDL (42 µL, ~0.04 mmol) was added for the reaction for 3.5 h. DMPA (2.02 g, 0.0154 mol) dissolved in 12 mL DMF was added into the reaction solution for a 2 h reaction at 85 °C. The reaction temperature was reduced to 60 °C. 1,4-BDO (0.57 mL, 6.4 mmol) and HEMA (1.1 mL, 9 mmol) were added to the system for chain expansion, and the WPU prepolymer with double bond terminals was generated by a constant temperature reaction for 4 h. After the temperature was reduced to 40 °C, TEA (2.13 mL, 15.4 mmol) was added for a neutralization reaction of 30 min. The carboxylic groups in DMPA were neutralized into carboxylic ions, and the hydrophilic ends of the chain segments were exposed. The stirring speed was adjusted to 2000 r/min, and 65 mL ultrapure water was added to the system for stirring for 15 min. A 10 wt.% APS solution (350 µL, ~0.01 mmol) was added to the mixture solution, and emulsion polymerization occurred for 3 h at 85 °C with a rotating speed of 300 r/min. A silane-modified WPU emulsion was obtained and stored in a tightly closed glass flask at 4 °C.

The prepared WPU solution was evenly spread on a Teflon plate and dried overnight at 60 °C. Transparent WPU films were obtained. By analyzing the infrared absorption spectrum of the film, we found a strong absorption peak at 2270 cm^{-1} in Figure S2, corresponding to the vibration absorption of -NCO. After the reaction, the absorption peak of WPU at 2270 cm^{-1} in the infrared spectrum disappeared, indicating that all the -NCO groups participated in the reaction. Moreover, the synthesized WPU films had an obvious absorption peak at 1026 cm^{-1}, corresponding to the vibration absorption of Si-O-Si; the -NH wide stretching band at 3325 cm^{-1} corresponded to the hydrogen bond interaction between -NH and C=O in the carbamate group. The results indicated that silane-modified WPU was successfully synthesized.

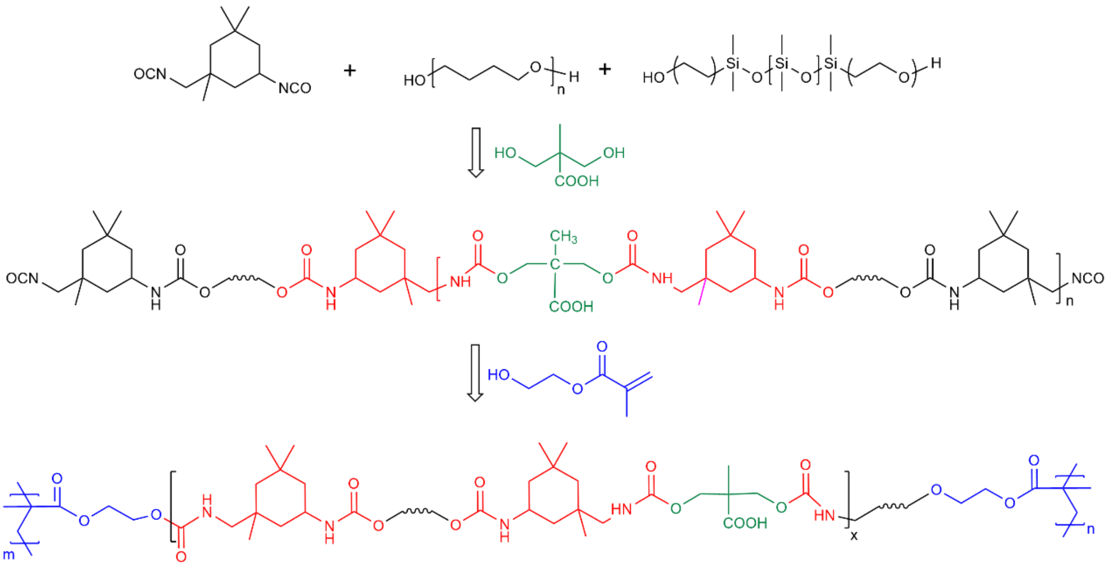

Scheme S1. Synthesis of WPU.

Preparation of WPU-IL$_x$ ionogels: WPU aqueous solution (30 wt.%) and [EMIM][DCA] aqueous solution (30 wt.%) were mixed in different volume ratios in a glass flask under stirring to obtain a mixed solution of WPU/IL$_x$. Then, the WPU/IL$_x$ mixture solution was cast onto a polyfluortetraethylene plate. WPU/IL$_x$ ionogels were finally obtained by evaporating the solvent at room temperature for 12 h.

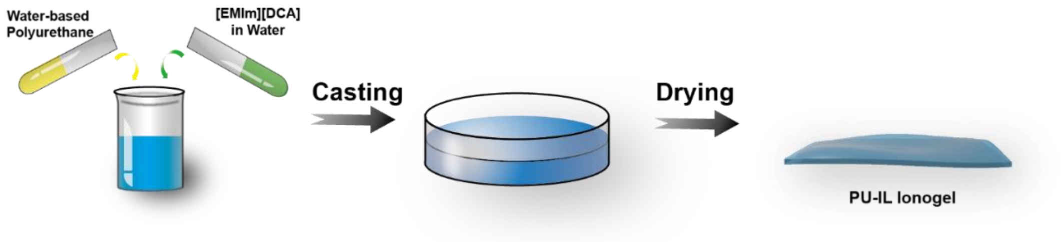

Scheme S2. Preparation of WPU/IL$_x$ by deposition techniques.

General test:

FTIR spectra were recorded on a Nicolet is10 (Thermo Fisher Scientific, China) using the attenuated total reflectance (ATR) method. Dynamic light scattering (DLS; Mastersizer2000/MAL1012737, Malvern panalytical, UK) was used to measure the average diameter of the WPU/IL$_x$ colloidal complex. Differential scanning calorimetry (DSC) measurements were performed on a DSC214 instrument (Netzsch, Germany) from -100 to 150 °C with a heating rate of 10 °C/min. Digital images were captured using a Nikon D5100 camera (Nikon, Japan). X-ray diffraction (XRD) tests were conducted on a Bruker-axs XRD. Electromyography (EMG) signals were collected by a BMD101 EMG Bluetooth module

(NeuroSky, USA). Optical loss was texted by an optical detector (QE65 PRO, Ocean optics). Optical loss was calculated as follows:

$A(\lambda) = 10\, lg\left(\frac{1}{P(\lambda)}\right) * 0.02/d$ .... (1)

where $A(\lambda)$ is the optical loss of the ionogels with an equivalent thickness of 0.2 mm, $P(\lambda)$ is the transmittance, and d is the thickness of the ionogels.

Mechanical characterization:

The ionogels were cut into dumbbell-shaped samples. The lengths of all samples were controlled at 1 cm, and their thickness and width values were measured with a micrometer. Mechanical tests were conducted on a microtester 5948 (Instron, America), including uniaxial tension tests, stress relaxation tests, loading–unloading tests, and cyclic loading tests. If not specified, the displacement rate of 10 mm/min was adopted for the tests**.**

Because of Cyclic tensile tests in 50 cycles at a strain of 100% take too long time we increase the stretching rate of 100mm/min.

Poisson's ratio test:

The ionogels was cut into long strips, which were stretched 10% using a microtension meter; the longitudinal sizes before and after stretching were measured to obtain the longitudinal elongation using a Vernier caliper. According to the equation,

$\varepsilon_x = -\nu\varepsilon_y$ .... (2)

where ε_x is the longitudinal tensile ratio, ν is Poisson's ratio, and ε_y is the transverse tensile ratio. Poisson's ratio ν was calculated and is listed in Table 1.

WPU/IL$_x$ ionogel conductivity tests:

The ionogel was cut into a cuboid for the measurement of conductivity by the following equation:

$\sigma = \frac{L}{RA}$ .... (3)

where σ is conductivity, R is ionogel resistance, L is the ionogel length, and A is the cross-sectional area. The size of the ionogel cuboid was measured by a Vernier caliper. Two stainless steel sheets that were much larger than the cross sections of the ionogels were attached to the bottoms of the ionogels to measure the resistance R values with an impedance analyzer (TH2839, Tonghui, China) in AC mode with the frequency of 1 kHz for all experimental tests.

Measurements of Nyquist plots:

Two identical pieces of circular stainless steel sheets were attached on each bottom of a cylinder ionogel to form a sandwich structure. The diameter of the stainless steel sheet was 20 mm, and the circular stainless steel sheets had the same areas as the cylinder ionogels. Before the test, 2 circular stainless-steel sheets were fixed with a clamp to ensure adequate contact of the ionogel with the stainless-steel sheet. The thickness of the ionogel was 0.2mm when clamped. The Nyquist plots were obtained by an electrochemical workstation (Chenhua chi660e) at a frequency of 10^5~1 Hz and an amplitude of 5 mV.

WPU/IL$_x$ ionogels resistive sensor tests:

The relative resistance changes ($\Delta R/R_0$) were calculated by an impedance analyzer (TH2839, Tonghui, China). $\Delta R/R_0$ was calculated as follows:

:$\frac{\Delta R}{R_0} = \frac{R - R_0}{R_0}$, ……………………………………………………………..(4)

where R_0 and R are the resistance values of the ionogels before and after stretching.

The gauge factor (GF) values of the ionogels were calculated as follows:

$GF=\frac{\Delta R/R_0}{\varepsilon}$, ………………………………………………………………………… (5)

where ε is the ionogel strain.

To investigate the resistance–temperature relationship, we placed the ionogels in the refrigerator and oven to record the resistance values using a desktop multimeter (DAQ 6510, Keithley, USA) in DC mode.

The low detection limit of the ionogels were calculated as follows:

$D = 3N/S$……………………………………………………………………………….. (6)

where D is the low detection limit of the gel sensor, N is the standard deviation of the $\Delta R/R_0$ signal generated by ionogels without stretching, and S is the sensitivity. D was calculated as 0.68%.

Humidity stability testing of ionogels:

We prepared saturated lithium bromide (6%RH), potassium acetate (23%RH), potassium carbonate (43%RH), sodium chloride (75%RH) and potassium sulfate (97%RH) solutions to obtain different humidity gradients by taking advantage of the different saturated vapor pressures of different saturated salt solutions. The resistance of the ionogels under specific humidity was recorded until the signal is stable. We recorded the resistance values using a desktop multimeter (DAQ 6510, Keithley, USA) in DC mode.

Conductivity tests of the ionogels under dynamic conditions:

According to Pouillet's Law, the resistance of an ionotronic ionogel (R) was expressed as follows[S1]:

$$R = \frac{L}{\sigma A}$$

where σ is the ionic conductivity, L is the length, and A is the cross-sectional area.

For a constant volume ($V = L \times A = L_0 \times A_0$) for ideal elastomers, the ionic conductivity change was calculated as follows:

$$\frac{\sigma}{\sigma_0} = \frac{\lambda^2}{R/R_0}$$

where σ_0 and R_0 are the initial conductivity and resistance, respectively, and the deformation ratio, λ, is defined as $\lambda = L/L_0$. Note that $\lambda = \varepsilon + 1$ (ε, strain). We calculated the conductivity changes σ/σ_0 of ionogels under strain elongations of 3%, 5%, 10%, 30%, 50% and 100%, and the results are recorded in Figure S11. As the ionic gel was stretched, the conductivities of the ionogels increased gradually from 102.8 to 174%.

Monitoring steps of the fingers, wrists, elbows, thighs and knees of a person:

First, plastic wrap was wrapped around the joint for monitoring where the ionogel would be attached. This location was chosen to prevent the human body from acting as a conductor to interfere with the detection of the ionogel. Then, the two ends of the ionogel were fixed to the surface of the body with plastic wrap. Additionally, wires secured by plastic wrap were used to connect the ionogel detector with an impedance analyzer (TH2839, Tonghui, China) to record real-time resistance changes caused by joint bending.

Monitoring steps of the heartbeat of the person:

When the heart beats, the left side of the chest moves up or down accordingly. A similar method to monitor the joint was used to attach a piece of ionogel to the chest using complete plastic wrap instead of using plastic wrap attached on both ionogel ends. Because of the very low fluctuation of the heart, the subject needed to hold their breath during the test to prevent the chest fluctuation caused by heaving.

A healthy male subject (the first author of this communication) was recruited from Xiamen University and provided written informed consent before participating in the study. The deformation characteristics of various parts of the human body were monitored in real time.

Simulation of resistive sensor test behavior:

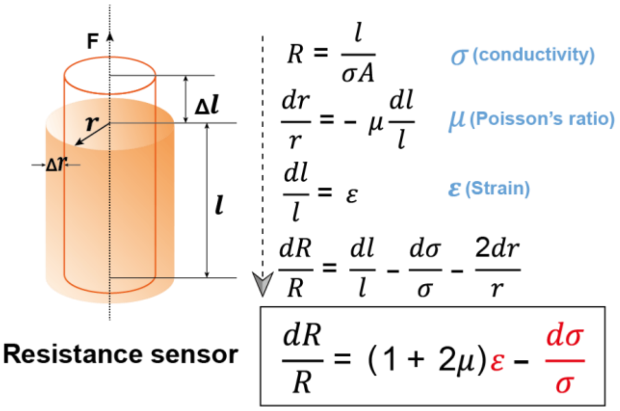

The sensing behavior of a resistive strain sensor was described by Equation (1). The GF of the sensor is positively correlated with the strain (ε), but negatively correlated with the change rate of the conductivity ($d\sigma$). However, as the strain increases, the conductivity gradually increases (Figure S12). This leads to a change in conductivity that will decrease the GF of the sensor. Through the equation, we can clearly find that the negative effect of the conductivity change rate can be reduced by increasing the initial conductivity (σ).

Fabrication of capacitive sensor:

Silk fluff and dirt were removed from the surface of the cocoon, and the cocoons were cut into pieces [S2]. Five grams of chopped cocoons were placed into 1 L of deionized water dissolved with 5 g of sodium bicarbonate and washed three times to remove silk sericin. The obtained silk fibroin fiber was immersed in 1 L of boiled deionized water to wash the sodium bicarbonate on the surface of the silk fibroin fiber three times. Finally, the silk fibroin fibers were dried in an oven at 60 °C for one day.

Five grams of silk fibroin fibers were dissolved in 30 mL of 9.3 mol/L lithium bromide solution at 60 °C for 2 h. The obtained silk fibroin solution was dialyzed using a dialysis tube with a molecular weight cut off of 3500 D. The deionized water for soaking was changed every 2 hours on the first day and every 4 hours on the second day. The obtained silk fibroin solution with a mass fraction of 6-7 wt.% was further concentrated in a dialysis tube (molecular weight cut off: 3500 D) using a 20 wt.% polyethylene glycol (molecular weight: 20000) solution. After 6–12 h of anti-dialysis, a silk fibroin solution of 15-17 wt.% was obtained and stored in a refrigerator at 4 °C.

The silk fibroin solution was poured onto the customized microneedle template. After defoaming by decompression, the silk fibroin solution was dried in a constant temperature and

humidity box (25 °C, 65% RH) for 24 h, and the silk fibroin microneedle was obtained after demolding and trimming.

The silk fibroin microneedle was used as a dielectric layer to be directly covered by polyurethane ionogel on both sides; then, copper foils were used to cover the surface of the ionogel layer to obtain a capacitive sensor. The capacitive sensor was connected by copper wire to an impedance analyzer (TH2839, Tonghui, China) for signal collection.

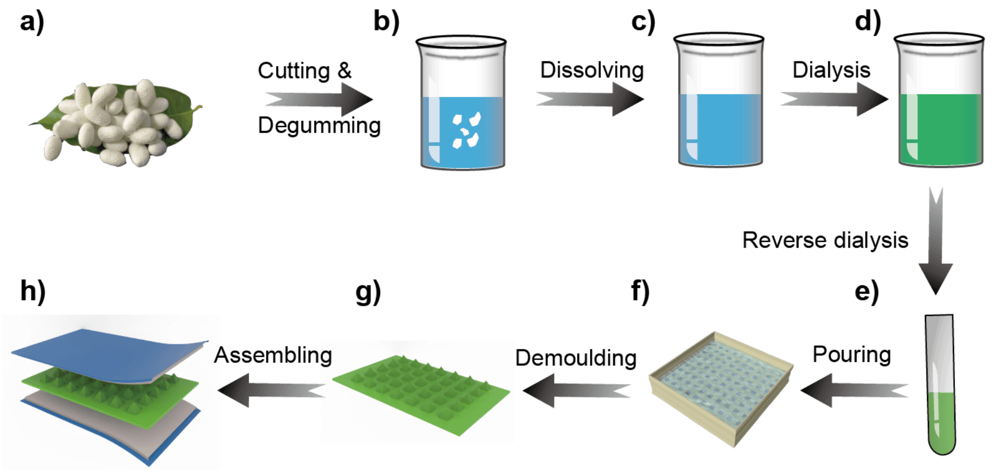

Scheme S3. Fabrication of capacitive sensors.

Tests of WPU/ILx ionogel capacitive sensor:

A capacitive sensor was fabricated by sandwiching a dielectric layer of silk fibroin film with microneedles between two $WPU/IL_{2.0}$ ionogel layers. To obtain the relationship between capacitance and pressure, the capacitive sensor was pressed by a microtester (5948, Instron, America) at a displacement rate of 1 mm/min; the capacitance value was monitored simultaneously by an impedance analyzer (TH2839, Tonghui, China).

Fabrication of EMG electrode:

The $WPU/IL_{2.0}$ ionogel was cut into a 1*1 cm square, which was connected by a copper wire to obtain a homemade electrode. The right arm flexure motions of the volunteers were measured with a homemade electrode and a commercial Ag/AgCl electrode with glue.

Testing the SNR of the EMG electrode

The SNR of the EMG electrode was calculated as follows:

$$SNR = \frac{S_{(EMG\ Amplitude)}}{S_{(Baseline\ Noise\ Amplitude)}} \quad \ldots\ldots\ldots\ldots\ldots\ldots\ldots\ldots\ldots\ldots\ldots\ldots\ldots\ldots\ldots\ldots. (7)$$

We took the midline of the EMG signal at rest as the baseline and calculated the peak area of the action potential ($S_{(EMG\ Amplitude)}$), and the peak area of the resting potential at the same time ($S_{(Baseline\ Noise\ Amplitude)}$). The SNR was equal to the ratio of the two areas.

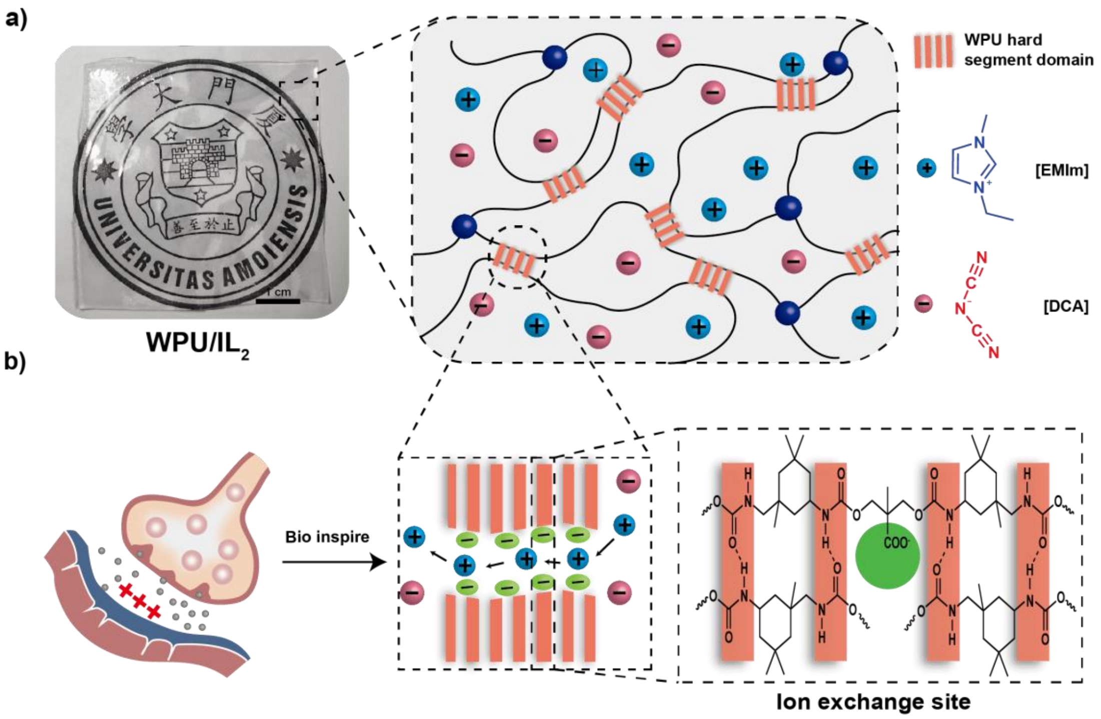

Figure S1. a) Optical photo and molecular network structure of WPU/$IL_{2.0}$. b) Conductive enhancement mechanism by introducing ion exchange sites.

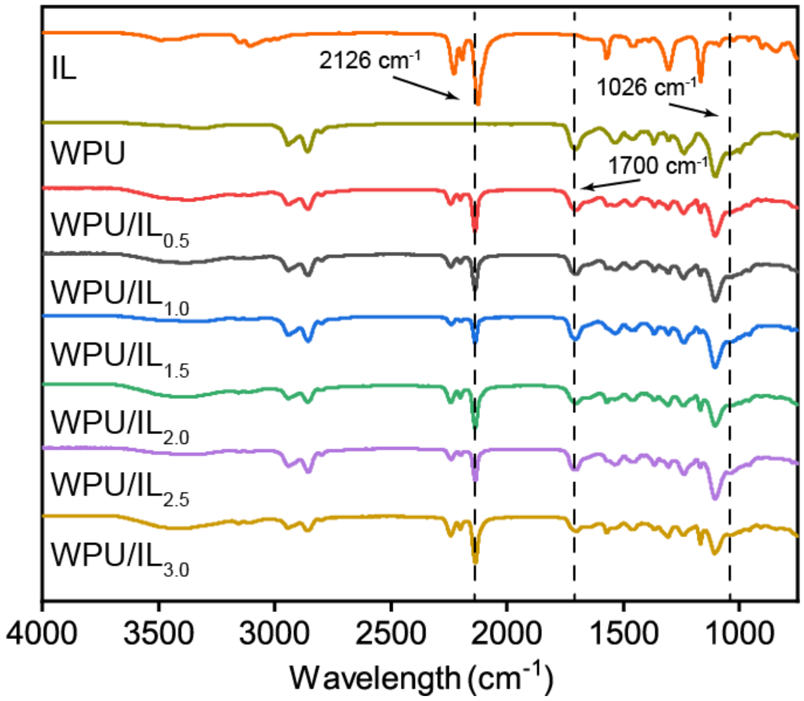

Figure S2. FTIR spectra of the IL, WPU film and WPU/IL$_x$ ionogels. The absorption peak at 1026 cm^{-1} corresponds to the vibration absorption of Si-O-Si, and the absorption peak at 1700 cm^{-1} represents the vibration absorption of C=O. These two sites are found only in the FTIR spectra of the WPU film and ionogels, while the anion symmetric stretching vibration of [DCA]$^-$ corresponding to the 2126 cm^{-1} site is found only in the FTIR spectra of the IL and ionogels.

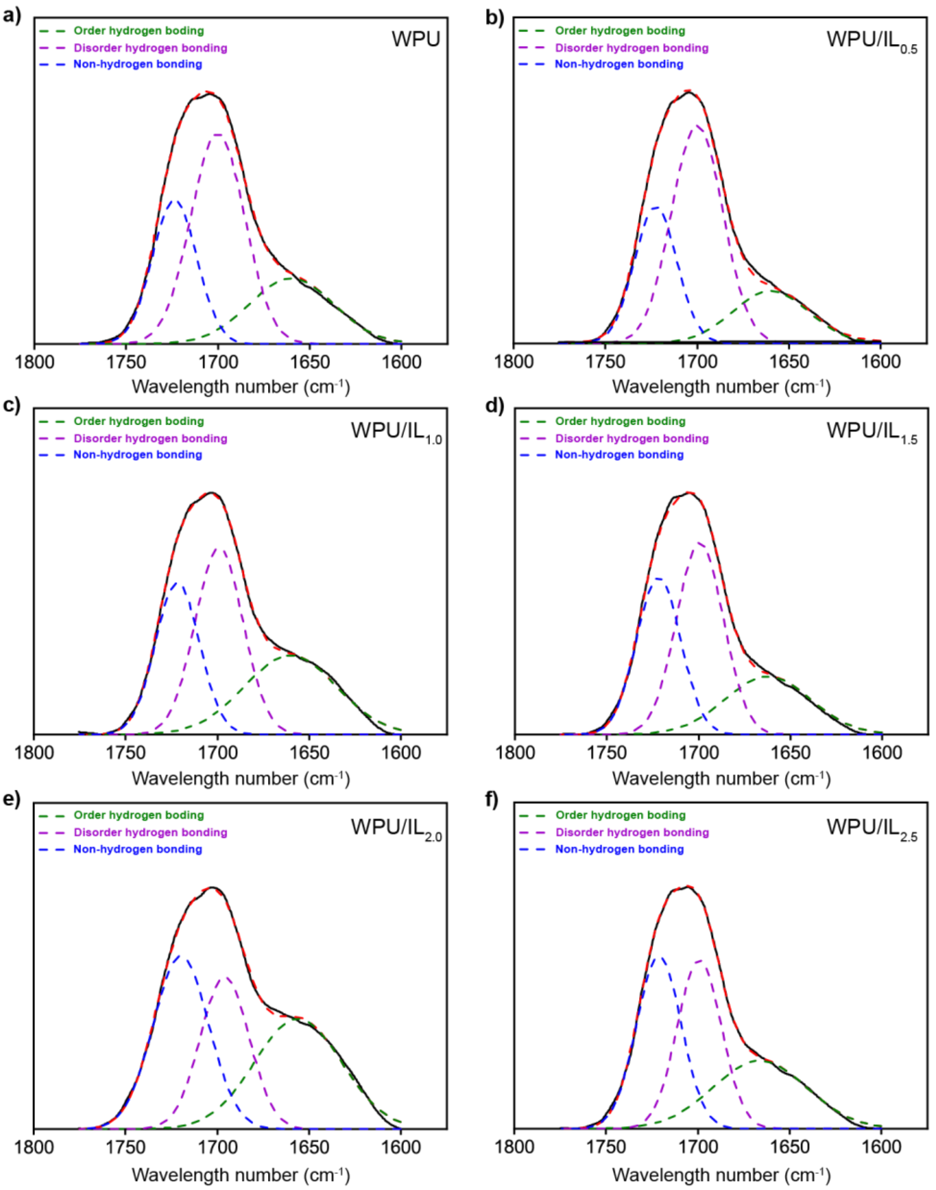

Figure S3. FTIR peak division of the vibration absorption peak of C=O of WPU/IL$_x$ at 1580–1775 cm^{-1} through the peak splitting method of deconvolution[S3,4].

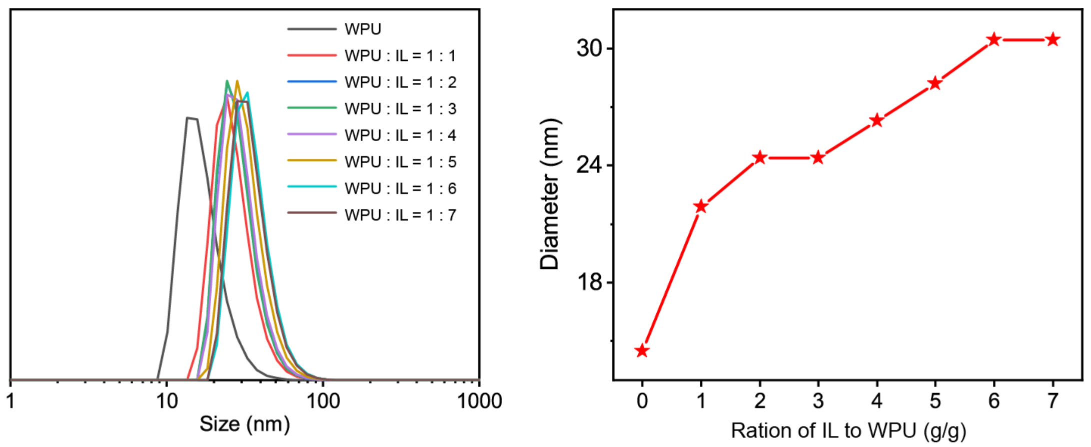

Figure S4. Average diameters of WPU/IL$_x$ colloids.

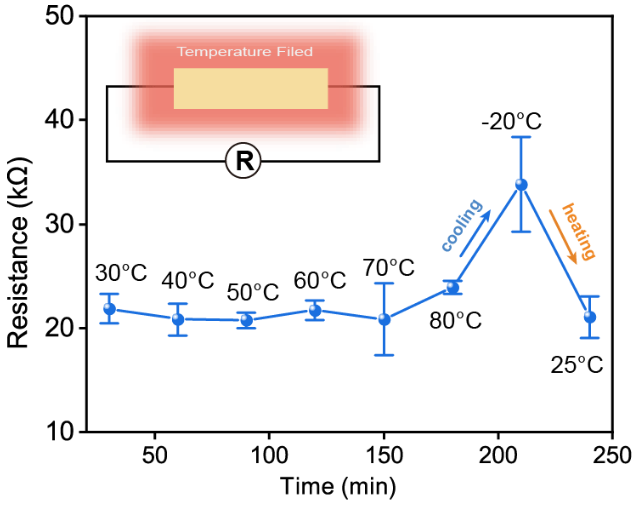

Figure S5. Resistance curve of WPU/IL$_{2.0}$ with temperature.

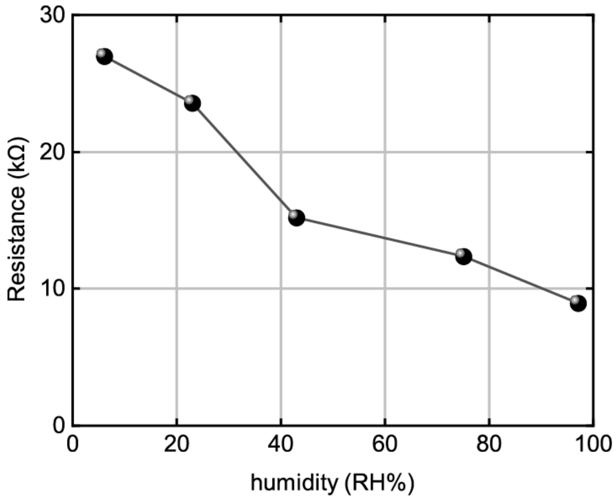

Figure S6. Resistance curve of WPU/IL$_{2.0}$ with humidity.

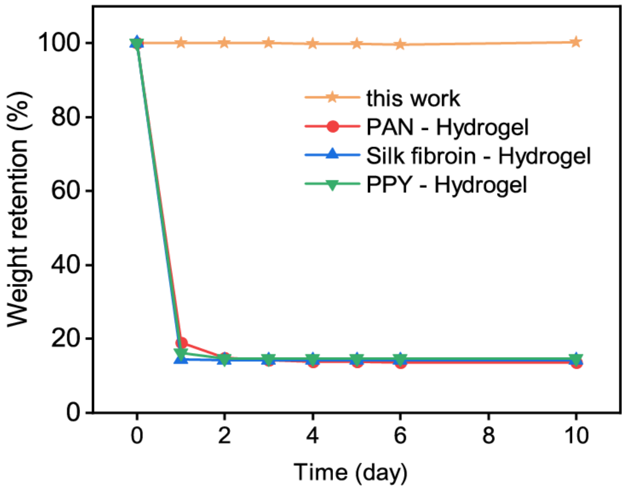

Figure S7. Water retention test of ionogels, polyacrylonitrile (PAN), silk fibroin and polypyrrole (PPY) hydrogel. The synthesis method of PAN[S5], silk fibroin[S6] and PPY hydrogel[S7] are from the literature.

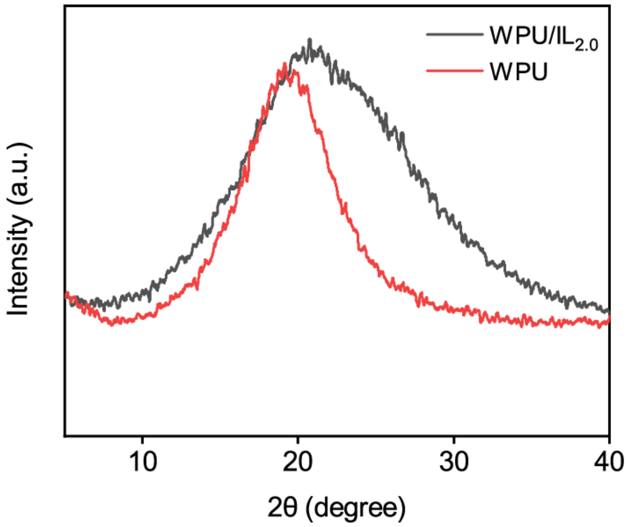

Figure S8. XRD spectra of WPU/$IL_{2.0}$ ionogels.

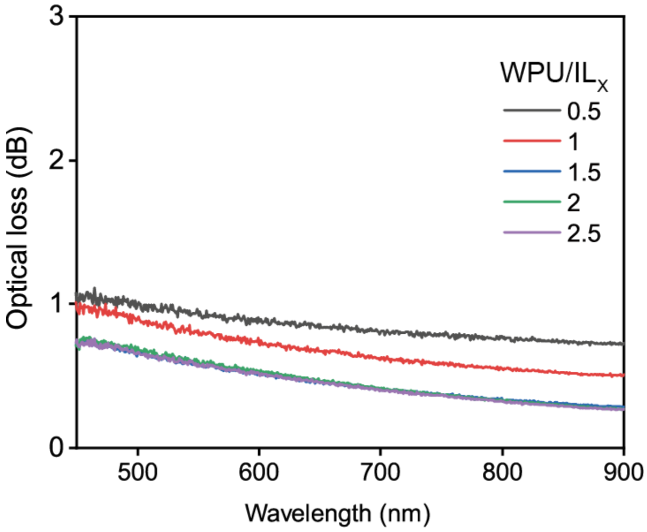

Figure S9. Optical losses of WPU/ILx ionogels.

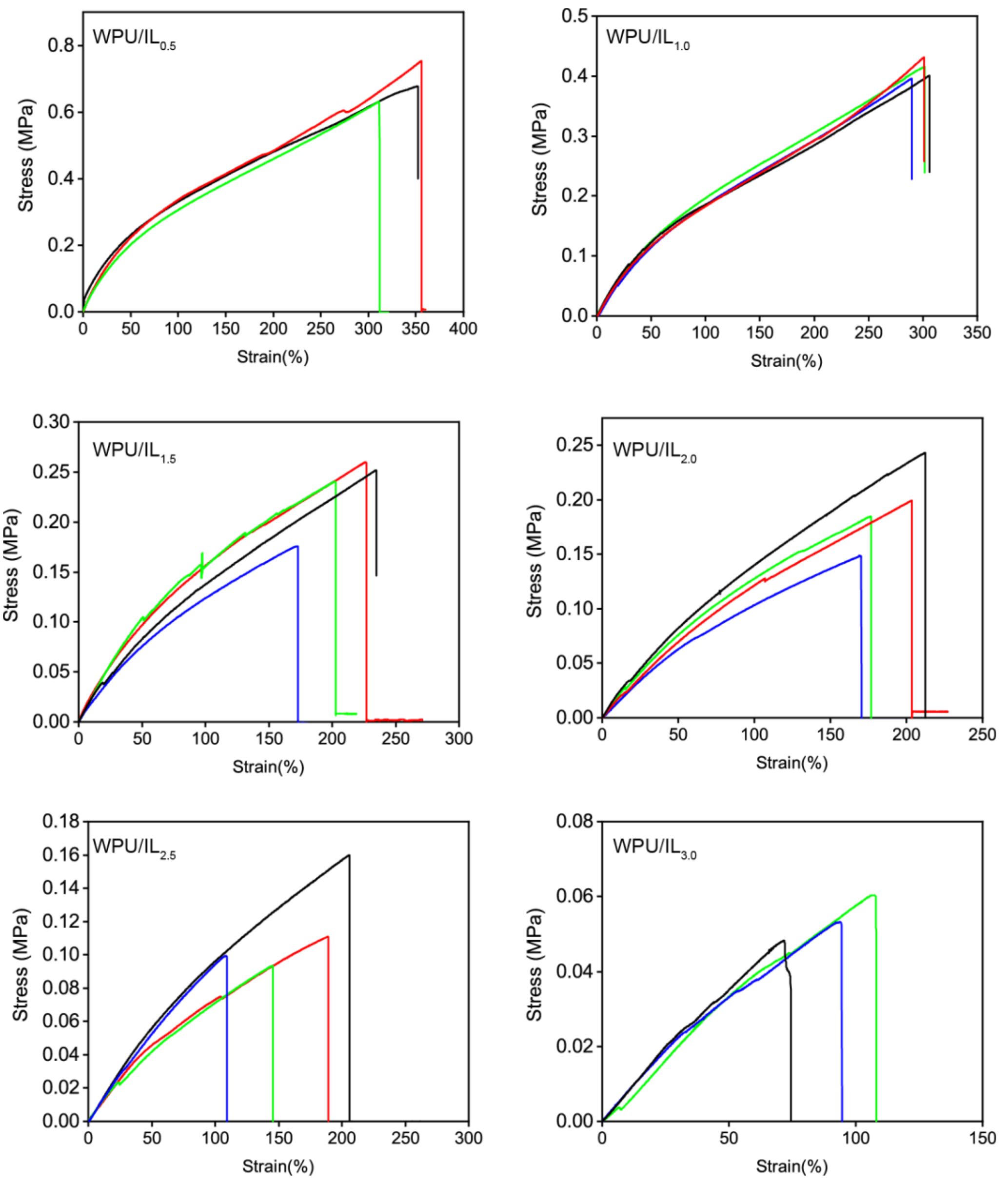

Figure S10. Stress–strain curves of WPU/IL$_x$ ionogels.

Table S1. Mechanical properties of WPU/IL$_X$ ionogels, including stretchability, Young's modulus, fracture strength, fracture toughness and Poisson's ratio.

WPU/IL$_X$ ionogels	Stretchablity (%)	Tensile strength (MPa)	Young's modulus (MPa)	Toughness (10^4 J/m^3)	Poisson's ratio
WPU/IL$_{3.0}$	92.49 ±[a] 16.83	0.054 ± 0.006	0.075 ± 0.006	0.029 ± 0.008	0.81 ± 0.19
WPU/IL$_{2.5}$	162.45 ± 43.60	0.116 ± 0.030	0.107 ± 0.010	0.113 ± 0.059	0.87 ± 0.14
WPU/IL$_{2.0}$	190.72 ± 19.73	0.194 ± 0.039	0.176 ± 0.035	0.216 ± 0.062	0.97 ± 0.14
WPU/IL$_{1.5}$	209.36 ± 27.29	0.235 ± 0.039	0.225 ± 0.036	0.295 ± 0.080	0.61 ± 0.09
WPU/IL$_{1.0}$	299.75 ± 7.26	0.410 ± 0.015	0.298 ± 0.020	0.702 ± 0.034	0.83 ± 0.13
WPU/IL$_{0.5}$	340.42 ± 24.36	0.687 ± 0.060	0.586 ± 0.040	1.417 ± 0.209	0.76 ± 0.16

[a] Variance was calculated from parallel samples.

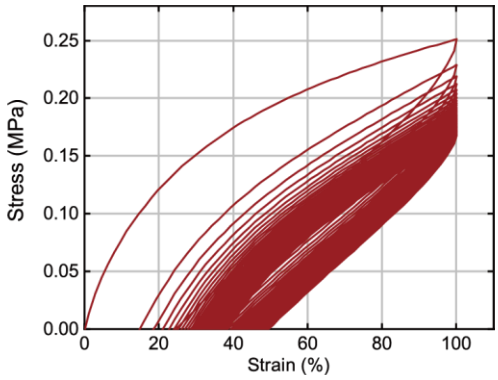

Figure S11. WPU/IL$_{2.0}$ ionogels' cyclic tensile tests in 50 cycles at a strain of 100%.

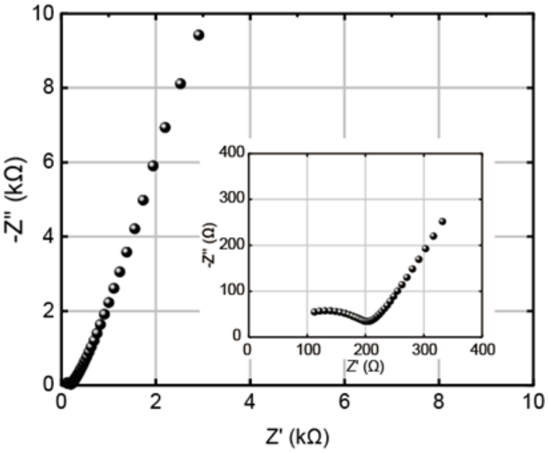

Figure S12. AC impedance spectra of ionogels.

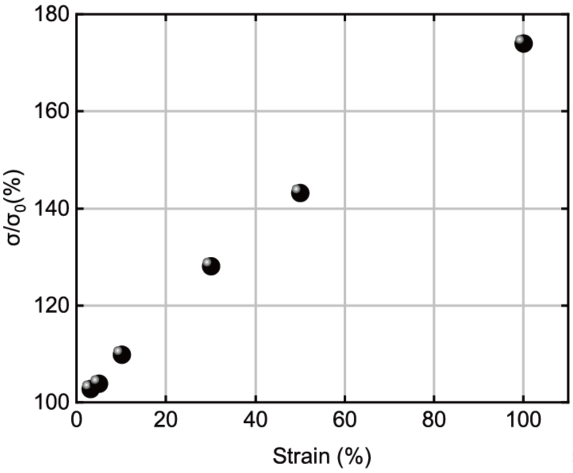

=====

Figure S13. Conductivities of the ionogels at dynamic conditions.

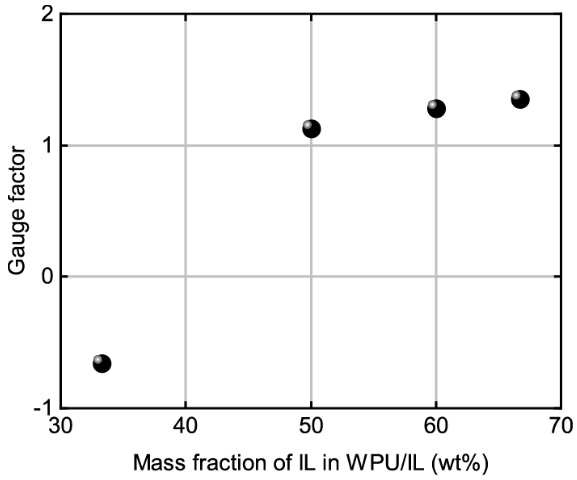

Figure S14. GF values of WPU/IL_x ionogel sensors with different IL contents.

[S1] M. Yao, B. Wu, X. Feng, S. Sun, P. Wu. *Adv. Mater*. **2021**, *33*, 2103755.

[S2] Z. F. Lin, Y. R. Li, G. Q. Meng, X. L. Hu, Z. C. Zeng, B. C. Zhao, N. B. Lin, X. Y. Liu. *Biomacromolecules* **2021**, *22*, 5319.

[S3] J. Wang, L. Ye. *Compos, Part B-Eng* **2015**, *69*, 389.

[S4] Y. T. Han, J. L. Hu, Z. Y. Xin. *Prog. Org. Coat.* **2019**, *130*, 8.

[S5] C. X. Yin, L. Weng, Z. X. Fei, L. Y. Shi, K. K. Yang. *Chem. Eng. J.* **2022**, *431*, 134206.

[S6] Z. W. Chen, H. H. Zhang, Z. F. Lin, Y. H. Lin, J. H. van Esch, X. Y. Liu. *Adv. Funct. Mater.* **2016**, *26*, 8978.

[S7] M. Yang, X. N. Ren, T. T. Yang, C. Xu, Y. Q. Ye, Z. W. Sun, L. H. Kong, B. Wang, Z. Q. Luo. *Chem. Eng. J.* **2021**, *418*, 129483.
